# Selective Decontamination of the Digestive Tract Reduces Pneumonia and Mortality

**DOI:** 10.1155/2010/501031

**Published:** 2010-10-07

**Authors:** Lenneke E. M. Haas, Marcus J. Schultz

**Affiliations:** ^1^Department of Intensive Care Medicine, Academic Medical Center, University of Amsterdam, Meibergdreef 9, 1105 AZ Amsterdam, The Netherlands; ^2^Laboratory of Experimental Intensive Care and Anesthesiology (L.E.I.C.A), Academic Medical Center, University of Amsterdam, Meibergdreef 9, 1105 AZ Amsterdam, The Netherlands

## Abstract

Selective decontamination of the digestive tract (SDD) has been subject of numerous randomized controlled trials in critically ill patients. Almost all clinical trials showed SDD to prevent pneumonia. Nevertheless, SDD has remained a controversial strategy. One reason for why clinicians remained reluctant to implement SDD into daily practice could be that mortality was reduced in only 2 trials. Another reason could be the heterogeneity of trials of SDD. Indeed, many different prophylactic antimicrobial regimes were tested, and dissimilar diagnostic criteria for pneumonia were applied amongst the trials. This heterogeneity impeded interpretation and comparison of trial results. Two other hampering factors for implementation of SDD have been concerns over the risk of antimicrobial resistance and fear for escalation of costs associated with the use of prophylactic antimicrobials. This paper describes the concept of SDD, summarizes the results of published trials of SDD in mixed medical-surgical intensive care units, and rationalizes the risk of antimicrobial resistance and rise of costs associated with this potentially life-saving preventive strategy.

## 1. Introduction

Potentially pathogenic microorganisms such as Gram-negative bacteria (including *Pseudomonas aeruginosa*), Gram-positive bacteria (including *Staphylococcus aureus*), and yeasts rapidly colonize stomach and intestines of critically ill patients [1]. Retrograde colonization of the oral cavity and throat may occur, and microaspiration into the lung could eventually result in pneumonia [2]. Prevention of colonization of oral cavity, throat, stomach, and intestines could reduce the incidence of respiratory tract infections, thereby improving outcome of intensive care unit (ICU) patients. 

 Selective decontamination of the digestive tract (SDD) is one strategy to prevent colonization of oral cavity, throat, stomach, and intestines of ICU patients. Numerous randomized controlled clinical trials have suggested SDD a beneficial strategy. Indeed, reductions in the incidence of pneumonia have been achieved with the use of SDD in critically ill patients [3]. However, only 2 trials showed SDD to reduce mortality [4, 5]. This may have caused caregivers to become indisposed to apply this strategy in daily practice. In addition, fear for emergence of antimicrobial resistance and escalation of costs associated with SDD, at least in part, hampered widespread implementation of this preventive strategy.

It should be noticed that the concept of colonization and infection as presented by Stoutenbeek and van Saene concerned trauma patients. It can be questioned whether this concept holds true for other ICU patients. Indeed, these patients are older, have significant comorbidities, and are frequently on antibiotics already at or before admission to the ICU.

We here describe the concept of SDD. We summarize the numerous clinical trials of SDD, focusing on trials applying the original SDD strategy in mixed medical-surgical ICUs and having pneumonia and/or mortality as a primary endpoint. Concerns over bacterial resistance and costs associated with SDD are discussed and rationalized.

## 2. ICU-Related Infections

### 2.1. Incidence and Outcome

ICU-related infections, in particular pneumonia, constitute a major problem during critical illness [6, 7]. Up to 50% of critically ill patients develop pneumonia [8]. When critically ill patients develop pneumonia, ICU and hospital mortality may double [9]. In accordance, patients with pneumonia need mechanical ventilation for a longer period of time and have a prolonged stay in ICU and hospital [10, 11]. Consequently, costs rise when pneumonia develops [12–14].

### 2.2. Primary and Secondary Endogenous versus Exogenous Infections

ICU-related infections can be classified into primary endogenous, secondary endogenous, and exogenous infections [15].

Primary endogenous infections are caused by pathogens carried in throat, stomach, and/or intestines of patient on ICU-admission. They occur generally within one week after admission and can be prevented by parenteral antibiotics administered directly after admission to the ICU.

Secondary endogenous infections may also occur soon after admission to the ICU. Contrary to primary endogenous infections, pathogens involved with secondary endogenous infections are not carried in throat, stomach, and/or intestines on admission but acquired during stay in ICU, and mostly from other patients via the hands of caregivers. Most of these infections could be banned if colonization is prevented. 

Exogenous infections can occur at any time during stay in ICU and occur when exogenous pathogens are accidentally introduced into a sterile internal organ without previous carriage.

### 2.3. Pathogenic Microorganisms of ICU-Related Infections

Micro-organisms differ in their pathogenicity. For example, fast majorities of ICU patients carry *Enterococcus* spp. in high concentrations in the intestines; infections caused by these microorganisms are rare. Conversely, 30%–40% of ICU patients who carry aerobic Gram-negative bacteria (including *P. aeruginosa *and* Klebsiella *spp.) in the oral cavity, throat or intestines develop an infection caused by these organisms. 

The pathogenicity can be expressed in the Intrinsic Pathogenicity Index (IPI) [16]. IPI is number of patients infected by species *x*/number of patients carrying species × in throat or intestines. The range of IPI is from 0 to 1. Carriage of a microorganism with an IPI close to 0 will seldom be followed by an infection. Carriage of a microorganism with an IPI close to 1 will almost always be followed by an infection. According to the IPI, microorganisms can be divided in low, potentially and highly pathogenic microorganisms (Table 1). Prevention of carriage with pathogens with an IPI close to 1 is thought to benefit ICU patients.

## 3. Prevention of ICU-Related Infections

### 3.1. Selective Decontamination of the Digestive Tract

Multiple strategies to reduce the incidence of respiratory infections in ICU patients have been evaluated, including SDD. Van der Waaij et al. were the first to describe the concept of SDD in 1971 [17]. The concept of SDD is based on colonization resistance—the intact, anaerobic intestinal flora is protective against secondary colonization with Gram-negative aerobic bacteria. Disturbance or loss of this anaerobic flora leads to increased colonization and increased risk of infection with facultative aerobic bacteria. SDD should prevent colonization with aerobic Gram-negative bacteria and other pathogens, without disrupting the anaerobic flora with the aim to reduce the incidence of secondary infections.

### 3.2. Components of SDD

**Figure 1:**
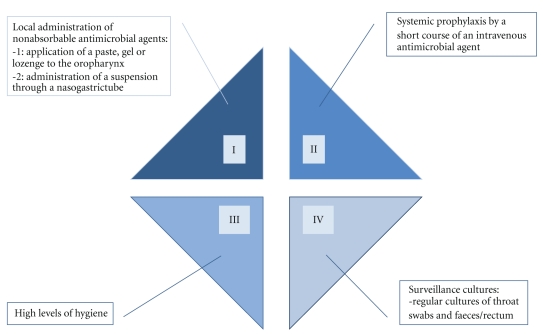
The 4 components of the original SDD regimen.

SDD classically consists of 4 components [18] (Figure 1):

- selective eradication of pathogenic microorganisms in the oral cavity and decontamination of the stomach and intestines by local administration of nonabsorbable antimicrobial agents—the first is reached by application of a paste, gel, or lozenge to the oropharynx, the second by administration of a suspension through a nasogastric tube;
- systemic prophylaxis by a short course of an intravenous antimicrobial agent—to prevent respiratory infections that may occur during the ICU stay, caused by commensal respiratory flora;
- high levels of hygiene to prevent cross-contamination;
- surveillance cultures (regular cultures of throat swabs and feces/rectum) to monitor the effectiveness of SDD.

It has been suggested that failure to apply this complete 4-component model reduces the effectiveness of SDD [19], although this has neither been tested nor proven.

## 4. Studies of SDD

### 4.1. Search Strategy

We identified relevant publications on SDD by searching the PubMed, EMBASE, and Cochrane Library databases using the following terms or MeSH subject heading: [intensive care], [critical care], [critical illness], [critical mortality], [infections], [pneumonia], [infection control], [selective digestive decontamination], [antibiotic prophylaxis], [antibacterial agents/therapeutic use], [bacterial infections/prevention and control], [drug resistance (microbial/bacterial)], [costs and cost analysis], [cost–benefit analysis], [cost-effectiveness analysis], [economics], [drug costs], and [health care costs]. Reference lists from identified citations and relevant review articles were hand searched for additional potentially relevant publications and abstracts. 

With this search strategy, trials analyzing the oral decontamination strategy were also found, but subsequently we focused on the complete SDD regimen. We also restricted our search to trials performed in mixed medical-surgical ICUs and to studies that had pneumonia and/or mortality as the primary endpoint. Adjacent to this, we looked for studies that focused on antimicrobial resistance induced by SDD and studies that analyzed costs of SDD treatment.

### 4.2. Search Results

The search yielded 336 manuscripts of potential interest. Studies that were performed in specific subgroups of ICU-patients, such as neurosurgical patients, liver transplant patients, burn patients, trauma patients, patients with severe pancreatitis, and patients after open-heart surgery, after gastrectomy or after oesophageal resection, were excluded. We also excluded studies of SDD in the pediatric ICU setting. We subsequently focused on mortality and pneumonia, antimicrobial resistance, and costs. This left us with 20 publications on trials (Table 2), and 15 meta-analyses (Table 3). addition, our search yielded several publications dealing with antimicrobial resistance (Table 4) and costs of SDD, respectively.

### 4.3. Study Heterogeneity

Although we focused on trials applying the classical 4-component SDD, studies remained heterogenic. 

Different methods of randomization were used, for example inclusion per time period or per ICU. There was also great variation in the number of patients included in the retrieved trials. 

Second, there was great diversity in used prophylactic antimicrobials. In the original design, SDD consisted of locally applied polymyxin E, tobramycin, and amphotericin B plus systemically applied cefotaxim [74]. In one trial locally applied amphotericin B was replaced by nystatin [27]. In other trials tobramyein was replaced by a quinolone [4, 23, 75], gentamicin [66, 76], or neomycin [27, 34]. In many trials, cefotaxim was replaced by another antimicrobial, such as ceftazidime [30], ceftriaxone [77], ciprofloxacin [56, 75], ofloxacin [33], or trimethoprim [4]. 

Third, criteria for pneumonia were very diverse. Indeed, clinical, radiological, and microbial criteria versus bronchoscopic techniques with quantitative cultures were used. Consequently, the incidence of VAP ranged from as low as less than 10% to as high as 85%. Also, great variation in the mortality of the control group was seen in the different trials, making comparison of studies more difficult.

## 5. The Effect of SDD on the Incidence of Pneumonia and Mortality

With the exception of 3 trials [25, 28, 31], a reduction of the incidence of pneumonia was seen with the use of SDD (Table 2). Reductions were larger in trials that used more loose criteria for pneumonia. It can be argued that in trials that used only clinical and/or radiologic criteria with or without positive cultures of tracheal secretions the reduction in respiratory tract infections was in fact a reduction in purulent bronchitis and not a reduction in pneumonia per se [3]. All meta-analyses demonstrated a reduction of the incidence of pneumonia with the use of SDD (Table 3).

There were only 2 small studies that demonstrated a reduction of mortality with the use of SDD [4, 5]. Notably, most studies were too small to show a significant effect of SDD on mortality (Table 2). Two recently published well-powered trials also show SDD to reduce mortality of ICU patients [36, 37]. With the exception of 1 meta-analysis [38], reduced mortality rates of ICU patients were seen with the use of SDD (Table 3).

## 6. Effects of SDD on Microbial Resistance

One concern with prophylactic use of antimicrobial agents is the risk of the emergence of resistant pathogens [78, 79]. Notably, in most trials colonization with resistant bacteria or an increase of superinfections was not reported (Table 4). One trial that specifically addressed the issue of microbial resistance found that resistance rates were actually higher in the control population than in the SDD-treated population [36]. In addition, a reduction in the incidence of multiresistant *Klebsiella* spp. with SDD use was seen in 3 other trials [65, 80, 81]. 

However, more recently it was shown that SDD was associated with a gradual increase of rates of ceftazidime-resistant Gram-negative bacteria in the respiratory tract [72]. The rate of resistant Gram-negative bacteria in the gastrointestinal tract significantly increased after discontinuation of SDD [72]. 

SDD may promote colonization with Gram-positive bacteria. The rate of colonization with Gram-positive strains was significantly higher, and more cases of Gram-positive bacteremia occurred in SDD-treated patients [33, 34]. 

Two meta-analyses showed that resistance against SDD antimicrobials is not emerging with long-term use [43, 50]. Use of SDD was even associated with a lower rate of colonization as well as infection with resistant Gram-negative bacteria [34, 65, 82]. 

Additional research is mandatory to determine whether SDD is a safe strategy with respect to the risk of emergence of antimicrobial resistance, especially in countries with higher endemicity of multidrug-resistant pathogens, because with the available evidence this risk cannot completely be denied.

## 7. Costs of SDD

Several studies compared costs of antimicrobial therapies between the SDD strategy and the control strategy, though in all these trials cost was only a secondary endpoint.

Costs have been calculated in different ways. One trial showed no differences in costs between the SDD strategy and the control strategy [67]. Studies that used cefotaxime showed a reduction of costs. Two other trials, however, showed higher costs with SDD when a quinolone was given for systemic prophylaxis [33, 75]. 

Four trials analyzed total ICU costs per survivor [5, 61, 77, 83]. These trials showed a reduction of costs with the use of SDD, which was the result of reductions in length of stay and reduced use of systemic antibiotics.

## 8. Discussion

The findings of our review of the literature can be summarized as follows: (I) numerous trials of SDD show a reduction of the incidence of pneumonia with this preventive strategy, (II) well-powered trials of SDD show a reduction of mortality with SDD, (III) although SDD is associated with induction of antimicrobial resistance in some studies, it certainly was not a problem in all trials, and (IV) SDD seems to be a cost-effective strategy.

Preventive measures against pneumonia in critically ill patients include, but may not be restricted to, early weaning from mechanical ventilation, hand hygiene, aspiration precautions, and prevention of contamination—at times summarized with the acronym “WHAP” [84]. It has been demonstrated that an educational initiative on WHAP, directed at respiratory care practitioners and ICU-nurses, was associated with decreases in VAP incidence rates of up to 61% [84]. One of the problems with the interpretation of the trials of SDD is that it is uncertain whether the caregivers complied with these prevention strategies. Indeed, only “high levels of hygiene” is a component of SDD.

Interpretation and comparison of the results of trials of SDD are complicated by the many dissimilarities amongst SDD regimens that were applied. We tried to solve this issue by focusing on studies that investigated the effect of the classical SDD regimen, applying all 4 components thought to be important for its efficacy [18]. Nevertheless, large differences remained present. Interpretation and comparison of the results of trials of SDD are also complicated by the difference in quality of the individual studies. For instance, large variation in the incidence of pneumonia was seen, due to difference in the way pneumonia was diagnosed. The incidence of pneumonia in studies that used bronchoscopic techniques with quantitative cultures was half of that in studies made the diagnosis of pneumonia on clinical and radiological criteria. Nevertheless, since all studies showed a positive effect of SDD with respect to the rate of pneumonia, we consider the evidence for SDD as an effective prophylactic strategy sufficient. Thereby it can be questioned if it is necessary to give the “full” protocol, with intestinal decontamination and systemic cefotaxime. In the light of the recently published work by de Smet et al. the role of intestinal decontamination and of systemic cefotaxime seems to be questionable [37].

The fast majority of trials of SDD showed no effect on mortality. It should be noted, however, that most studies were underpowered to show any effect on mortality. The last 2 trials of SDD, however, were adequately powered [36, 37]. Since these 2 trials showed SDD to reduce mortality of ICU patients, we also consider the evidence for SDD as a life-saving strategy sufficient. 

It can be questioned if SDD should be given to all ICU patients or restricted to selected groups. We have solely focused on the effect of SDD in patients in mixed medical-surgical ICUs. 

The emergence of antimicrobial resistance with SDD has been the subject of numerous hot debates [85–87]. It has been argued that the use of SDD would promote the growth of resistant bacteria. In theory, this is a potential adverse effect of SDD treatment, but in low antibiotic resistance endemic areas this seems not to be a problem. The 2 trials that investigated resistance were performed in The Netherlands, a country with the lowest use of outpatient antibiotics, generally a narrower spectrum antibiotic for hospitalized patients, a low incidence of methicillin-resistant *S. aureus*, vancomycin-resistant *Enterococci* (VRE) other multidrug-resistant pathogens, and extended spectrum bèta-lactamase (ESBL) producing pathogens [88, 89]. This situation is markedly different from other centers across the world.

Because SDD is not active against resistant Gram-positive bacteria, it may promote colonization with bacteria such as *S. Aureus*, and *E. faecalis *and it can lead to infections with these bacteria in critically ill patients [90–92]. Patients' illness causes conversion of carriage of normal to abnormal flora. Most ICU patients have bacterial overgrowth. The increased spontaneous mutations lead to polyclonality and antimicrobial resistance [93]. In this way, treatment with SDD could promote gut overgrowth of intrinsically resistant bacteria, such as MRSA and VRE, although with endemicity no increase has been found [36, 94, 95]. However, it cannot be excluded that in countries where VRE is endemic, SDD can have a negative effect on VRE. On the other hand, the reduced prescription of systemic-broad spectrum antibiotics in SDD-treated patients may also lead to decreased incidence of VRE. Although SDD does exert selection pressure on plasmid-mediated ESBL, emergence of ESBL-producing bacteria due to SDD has not been found. 

SDD seems a safe strategy with regard to the emergence of antibiotic resistance in low antibiotic resistance endemic areas, but with the available evidence, we cannot say that SDD is also a safe strategy in high endemicity areas. We should consider giving SDD only in low endemic areas till results in high endemic areas are available. Additional research is also mandatory to determine what to do when resistant pathogens do emerge. 

Another concern with SDD is the cost associated with this preventive strategy. Costs of SDD have been calculated in several studies, but most of these were not designed to analyze cost-effectiveness. The absence of studies on costs and cost-effectiveness is remarkable. It could be that a proper economic analysis in the ICU setting is difficult to perform, because it is hard to quantify the relative contribution of a single strategy. For example, the price of intravenous antibiotics can vary widely between hospitals, because the price is dependent on negotiations between local pharmacy and manufacturers. 

The most important cost component associated with SDD is the cost of antibiotics. As the aim of SDD is to reduce in ICU infections, including pneumonia, a reduction in total antibiotic use can be expected. Overall hospital costs may be lower, in part due to the decrease rate of pneumonia. Development of pneumonia is associated with up to 5 extra days of ICU treatment. Prevention of pneumonia substantially decreases the length of ICU stay and thus reduces costs per patient. 

## 9. Conclusion

Numerous randomized controlled trials have shown that SDD reduces the incidence of pneumonia. Two recently published well-powered trials also show SDD to reduce mortality of ICU patients. SDD can be associated with induction of antimicrobial resistance, but this seems not to be a clinical problem, at least not in countries with low endemicity. Finally, SDD seems to be a cost-effective strategy. Based on these findings, we favor the use of SDD in ICU patients.

## Figures and Tables

**Table 1:** Pathogenicity of microorganisms.

Site of carriage	Micro-organisms involved	Flora
*Low pathogenic microorganisms; IPI = 0.01*		
Throat	*Streptococcus viridans*	Normal
	*Veillonella* spp.	
	*Peptostreptococci*	
Gut	*Bacteroides* spp.	
	*Clostridium* spp.	
	*Enterococcus *spp	
	*Escherichia Coli*	
Vagina	Indigenous flora	
Skin	Coagulase-negative *Staphylococci *	

*Potentially pathogenic microorganisms; IPI = 0.3–0.6*		Normal
“Community” PPM		
Throat	*Streptococcus pneumoniae*	
	*Hemophilus. Influenzae*	
	*Moraxella catarrhalis*	
	*Staphylococcus aureus*	
	*Candida* spp.	
Gut	*Escherichia coli*	
	*Staphylococcus aureus*	
	*Candida* spp.	

“Hospital” PPM		

Throat and gut	*Klebsiella *spp.	Abnormal
	*Proteus *spp.	
	*Morganella *spp.	
	*Enterobacter *spp.	
	*Citrobacter *spp.	
	*Serratia *spp.	
	*Pseudomonas aeruginosa*	
	*Acinetobacter *spp.	
	Multiresistant *Staphylococcus aureus *	

*Highly pathogenic microorganisms; IPI = 0.9–1.0*		
“Epidemic” microorganisms		
Throat	*Neisseria meningitides*	Abnormal
Gut	*Salmonella* spp.	

See text for details.

**Table 2:** Incidence and relative risks of pneumonia and mortality in trials of SDD in mixed medical-surgical ICUs.

Publication	Year	*N*	Incidence of pneumonia (%)	ICU Mortality (%)
			SDD versus control (RR [95% confidence interval])	
Ledingham [20]	1988	324	3 versus 9%, *P* = .006	24 versus 24%, ns
Ulrich [4]	1989	100	6 versus 44%, *P* < .00001	31 versus 54%, *P* < .02
			(0.28 [0.13–0.59])	(0.69 [0.47–1.02])
Godard [21]	1990	181	2 versus 15%, *P* < .05	12 versus 18%, ns
McClelland [22]	1990	27	7 versus 50%, *P* < .05	60 versus 58%, ns
Aerdts [23]	1991	56	6 versus 62%, *P* = .0001	12 versus 10%, ns
			(0.09 [0.01–0.60])	(0,71 [0.25–2.02])
Blair [24]	1991	256	48 versus 82%, *P* = .002	15 versus 19%, ns
			(0.33 [0.18–0.62])	(0.17 [0.49 – 1.28])
Finch [25]	1991	49	0.69 [0.23–2.01], ns	1.56 [0.88–2.77], ns
Gaussorgues [26]	1991	118	N.A.	1.00 [0.69–1.44], ns
Cockerill [27]	1992	150	5 versus 16%, *P* = .03	11 versus 19%, ns
			0.33 [0.11–0.99]	0.69 [0.34–1.38]
Hammond [28]	1992	239	26 versus 34%, *P* = .22	12 versus 12%, ns
			0.82 [0.51–1.34]	1.08 [0.70–1.67]
Jacobs [29]	1992	91	0 versus 9%, ns	39 versus 53%, ns
			0.13 [0.02–0.95]	0.62 [0.37–1.05]
Rocha [5]	1992	101	15 versus 46%, *P* < .001	21 versus 44%, *P* < .05
			0.32 [0.15–0.68]	0.70 [0.49–1.02]
Winter [30]	1992	183	3 versus 18%, *P* < .05	36 versus 43%, ns
			0.18 [0.05–0.59]	0.83 [0.58–1.19]
Ferrer [31]	1994	80	18 versus 24%, ns	31 versus 27%, ns
			0.70 [0.33–1.46]	1.21 [0.63–2.34]
Palomar [32]	1997	129	17 versus 50%, *P* = .005	24 versus 31%, ns
			0,39 [0,21–0,73]	0,98 [0,52–1,84]
Verwaest [33]	1997	440	9 versus 18%, *P* = .026	18 versus 17%, ns
			0.53 [0.34–0.89]	1,17 [0.81–1.71]
Sánchez-García [34]	1998	271	11 versus 29%, *P* < .001	39 versus 47%, ns
			0.57 [0.40–0.81]	0.84 [0.63–1.11]
Parra Moreno [35]	2002	306	5 versus 20%, *P* < , 0001	N.A.
			0.30 [0.16–0.53]	
De Jonge [36]	2003	934	N.A.	15 versus 23%, *P* = .002
				0.65 [0.49–0.85]
De Smet [37]	2009	4035	N.A.	3.5% points absolute reduction, *P* = .02
				0.81 [0.69–0.94]

*N*: number of patients; ns: not significant; N.A.: not available.

**Table 3:** Odds ratios for pneumonia and mortality in meta-analyses of trials of SDD.

Publication	Year	*n*/*N*	Pneumonia	Mortality
			OR [95% confidence interval]	
Van den Broucke-Grauls [38]	1991	6/491	0.12 [0.08–0.19]	0.70 [0.45–1.09]
SDD Group [39]	1993	22/4142	0,37 [0.31–0.43]	0.90 [0.79–1.04]
Heyland [40]	1994	24/3312	0.46 [0.39–0.56]	0.87 [0,79–0.97]
Kollef [41]	1994	16/2270	0.15 [0.12–0.17]	0.02 [−0.02–0.05]
Hurley [42]	1995	26/3768	0.35 [0.30–0.42]	0.86 [0.74–0.99]
D'Amico [43]	1998	16/3361	0.35 [0.29–0.41]	0.80 [0.69–0.93]
	1999	21/N.A.	N.A.	0.70 [0.52–0.93]^a^
Nathens [44]				0.91 [0.71–1.18]^b^
Liberati [45]	2000	16/3361	0.35 [0,29–0.41]	0.80 [0.69–0.93]
Redman [46]	2001	N.A.	0.36 [0.28–0.46]	N.A.
Liberati [47]	2004	36/6922	0.35 [0.29–0.41]	0.78 [0.68–0.89]
Silvestri [48]	2007	51/8065	N.A.	0.80 [0.69–0.94]
Silvestri [49]	2008	54/9473	0.11 [0.06–0.20]	N.A.
Silvestri [50]	2009	21/4902	N.A.	0.71 [0.61–0.82]
Liberati [51]	2009	36/6914	0.28 [0.20–0.38]	0.75 [0.65–0.87]

^a^surgical patients; ^b^medical patients; *n*/*N*: number of trails/patients; OR: odds ratio; N.A.: not available.

**Table 4:** SDD and the emergence of antimicrobial resistance, in areas with high and low endemicity.

Endemicity	Main findings
MRSA	
High	Increase of colonization with MRSA [28, 31, 33, 52–55]
Low	No increase of colonization with MRSA [36, 56, 57]

VRE	
High	No increase of VRE infection rates [58, 59]; no increase of VRE infection rates when enteral vancomycin is added [26, 56, 60–64]
Low	No increase of VRE carriage [36]; increase of VRE isolates [57]

AGNB	
High	Decrease of multiresistant AGNB [54, 65]; lower incidence of carriage and infections with antibiotic resistant Gram-negative bacteria [36, 66–69]; no increase in prevalence of beta lactam- or aminoglycoside-resistant Gram-negative rods [57, 70]; increased antimicrobial resistance [5, 33, 71]
Low	Increased intestinal colonization with Gram-negative bacteria resistant to ceftazidime, tobramycin, or ciprofloxacin—discontinuation of SDD results in a rebound effect of ceftazidime resistant bacteria in the intestinal tract [72]; SDD increased the number of infections caused by multiresistant bacteria [73]

MRSA: methicillin-resistant *Staphylococcus aureus*, VRE: vancomycin-resistant *Enterococci *and AGNB: aerobic Gram-negative bacteria.
